# The biometric antecedents to happiness

**DOI:** 10.1371/journal.pone.0184887

**Published:** 2017-09-15

**Authors:** Petri Böckerman, Alex Bryson, Jutta Viinikainen, Christian Hakulinen, Mirka Hintsanen, Jaakko Pehkonen, Jorma Viikari, Olli Raitakari

**Affiliations:** 1 Turku School of Economics, Turku, Finland; 2 Labour Institute for Economic Research, Helsinki, Finland; 3 IZA, Bonn, Germany; 4 UCL Department of Social Science, London, United Kingdom; 5 NIESR, London, United Kingdom; 6 Jyväskylä University School of Business and Economics, Jyväskylä, Finland; 7 Department of Psychology and Logopedics, Faculty of Medicine, University of Helsinki, Helsinki, Finland; 8 Unit of Psychology, University of Oulu, Oulu, Finland; 9 Department of Medicine, University of Turku, Turku, Finland; 10 Division of Medicine, Turku University Hospital, Turku, Finland; 11 Research Centre of Applied and Preventive Cardiovascular Medicine, University of Turku, Turku, Finland; 12 Department of Clinical Physiology and Nuclear Medicine, Turku University Hospital, Turku, Finland; Xi'an Jiaotong University School of Medicine, CHINA

## Abstract

It has been suggested that biological markers are associated with human happiness. We contribute to the empirical literature by examining the independent association between various aspects of biometric wellbeing measured in childhood and happiness in adulthood. Using Young Finns Study data (n = 1905) and nationally representative linked data we examine whether eight biomarkers measured in childhood (1980) are associated with happiness in adulthood (2001). Using linked data we account for a very rich set of confounders including age, sex, body size, family background, nutritional intake, physical activity, income, education and labour market experiences. We find that there is a negative relationship between triglycerides and subjective well-being but it is both gender- and age-specific and the relationship does not prevail using the later measurements (1983/1986) on triglycerides. In summary, we conclude that none of the eight biomarkers measured in childhood predict happiness robustly in adulthood.

## Introduction

Happiness is beneficial to both individuals and society in many ways. Happier individuals are more resilient to illness and disease [1], survive longer [2] and are more productive at work [3]. Similarly, workplaces experiencing improvements in subjective well-being (SWB) improve their economic performance [4–5]. In philosophy hedonists promote the pursuit of happiness as a way to live “the good life”. In economics happiness is often equated with the utility individuals maximize [6]. It has been shown that individuals choose happiness over other goals, including life satisfaction and worthwhileness, most of the time (though health is of great concern as well) [7]. Yet, despite all this interest, it remains to a large extent unclear how happiness relates to physiological and biological aspects of well-being.

Biomarker or biological marker, refers to objective indicators of medical state such as blood pressure and pulse, which can be measured accurately and reproducibly. It is rare for studies to examine links between biomarkers and happiness. Instead the majority of the literature focuses on links between biomarkers and self-rated health. For example, Blanchflower and Oswald [8] show a positive association between BMI and the GHQ12 Mental Strain Index in the Health Survey for England. Oswald and Powdthavee [9] report similar correlations in the British Household Panel Survey. Some studies which supplement their investigations of self-rated health with other SWB measures tend to find biomarker correlations differ across SWB measures, even though various SWB measures are often positively correlated with self-rated health [10] and with one another [11]. For example, in their study of the elderly Rouch et al. [12] found higher triglyceride rates were associated with lower self-rated health seven years later in men, but not in women. However, association between triglycerides (i.e. the main constituents of body fat in humans) and life satisfaction was not found [12]. Moreover, using a longitudinal research design where nearly 500,000 participants from the UK Biobank were followed-up over 5 years, Ganna and Ingelsson [13] find biomarkers to be only poor predictors of mortality.

It is not surprising that particular biomarkers correlate in different ways with various SWB measures since they capture different dimensions of well-being [5, 14]. Happiness is a measure of positive affect akin to pleasure which is usually positively correlated with other SWB measures, but they are nevertheless conceptually distinct. The main reason why the literature linking biomarkers and happiness is so limited is the absence of large-scale data sets containing both. A previous study using data from the English Longitudinal Study of Ageing (ELSA), a nationally representative cohort of over 10,000 participants aged 50 years or older, showed that there is a negative association between happiness and pleasure and levels of circulating triglycerides [15]. The negative association with triglycerides was apparent for men and women, and it was also apparent with respect to eudemonia measures of SWB (sense of autonomy and purposeful engagement with life). These results were independent of demographic, socio-economic and health indicators. The only other biomarker that was similarly linked to positive psychological well-being among men and women was lung function. Other biomarkers were also associated with happiness, but results differed for men and women. In men, happiness was associated with smaller waist circumference and greater levels of dehydroepiandosterone sulfate whilst, among women, it was associated with lower concentrations of inflammatory markers (C-reactive protein and fibrinogen). The authors concluded that positive psychological well-being has biological correlates “that may be health protective, with distinctive patterns for men and women” [15].

In addition to ELSA there are also other data sources such as MIDUS (Midlife in the United States) data which could be used to examine longitudinal links between biomarkers and subjective well-being (see http://midus.wisc.edu/). However, to our knowledge, there are no prior empirical studies that have addressed the potential biomarker and subjective well-being linkage.

Although longitudinal data are available containing subjective well-being and biomarkers (i) these data are usually for older individuals [16]; (ii) their substantive focus tends to be healthy ageing, often with subjective well-being as a predictor of long-term health outcomes; (iii) they rarely contain biometric data collected in childhood; (iv) when there are childhood biometric data they are usually a small subset such as height and weight.

There are a number of reasons why one might anticipate an association between triglycerides and happiness. We know from other studies that triglycerides are linked to poorer physical health–notably a higher risk of metabolic syndrome, cardiovascular diseases, and type 2 diabetes mellitus [17]. High levels of triglycerides are also linked with other metabolic risk factors, such as elevated blood pressure, insulin resistance, and life-style variables including non-prudent diet and physical inactivity [18].

When considering the implications of Steptoe et al.’s [15] findings there are, perhaps, two issues worth bearing in mind. The first is that they identify a positive correlation between happiness and particular biomarkers in cross-sectional data. This makes causal inference difficult because it is hard to discern the direction of any causal linkage. In addition one cannot discount the possibility that common unobservable factors may determine both biometric and subjective well-being, thus inducing an omitted variables bias that might, conceivably, weaken or strengthen the observed cross-sectional correlation. For example, positive psychological activities are known to enhance happiness and may also alter biomarkers, such as those relating to the risk of disease [19].

The second issue, which is also relevant when considering the results presented by Rouch et al. [12], is that the analysis is conducted on older individuals. There is no way of knowing whether we can extrapolate from these results to younger cohorts. What we do know is that the age profile of most SWB measures is U-shaped, reaching its nadir in mid-life [20]. For example, in their study for the US, Oswald and Wu [10] find life satisfaction is at its lowest point aged 50 years. Analyses based on cross-sectional or short panel data on those in their middle years are therefore capturing SWB at a particularly low point in one’s life.

Other biomarkers in our data are thought to be related to SWB. For instance, there is cross-sectional evidence linking hypertension to lower life satisfaction [21]. In a closely related study Blanchflower and Oswald [8] documented that happiness is also correlated with lower levels of self-reported hypertension. Deaton and Arora [22] show that there is a positive relationship between concurrent height and SWB. Katsaiti [23] finds obesity has a negative effect on SWB in panel data for three countries: the instrumental variables strategy employed suggests the relationship is causal. There is also earlier Finnish evidence linking various measures of obesity to lower SWB [24]. But none of these studies use longitudinal research design linking biomarkers in childhood to adult happiness.

There are two fundamental reasons to expect persistent associations between biomarkers and happiness within individuals over time. There may be persistence in individuals’ biometric state such that one might anticipate a link between biomarkers in childhood and happiness in adulthood simply because the biomarkers in childhood are highly correlated with those in adulthood. Alternatively, biomarkers may vary within individuals across time but one’s biometric state in childhood might affect adult outcomes, in the same way as other childhood influences can have persistent effects. For example, there is evidence that bullying in school and one’s relationship with one’s parents have lasting psychological effects [25]. Clark and Lee [26] find several variables in childhood such as IQ score, parental income and parental education that are predictive of adult happiness but do not use biometric information in the analysis.

If there was persistence in the measures of biomarkers over time within individuals then one might get similar correlations between biomarkers and subjective well-being, whether one used contemporaneous measures or lagged biometric measures. This is an empirical matter, but one that is not easily tested since few studies collect biometric data on individuals over extended periods of time and, if they do, they are rarely collected in the same studies that contain subjective well-being on the same subjects over time. The advantage of longitudinal data is that it is easier to make causal inferences using the time sequencing of data than it is in the case of cross-sectional data since it is harder to discount the possibility of reverse causation when using cross-sectional data.

We contribute to the empirical literature linking how people say they feel and biomarkers of well-being by examining the independent association between biomarkers measured in childhood and happiness in adulthood. To our knowledge this is the first paper to do so. Thus, our paper makes an important methodological point regarding the direction of causality by using the time-sequencing in the data. Our data are from the Young Finns Study. We add additional controls from registers. The controls include age, sex, body size, family background, nutritional intake, physical activity, income, education and labour market experiences, as well as other relevant biomarkers measured in childhood.

## Materials and methods

### Data

To analyze the correlation between biomedical information and happiness we link data on biomarkers from the Cardiovascular Young Finns Study (YFS) to the Longitudinal Population Census (LPC) of Statistics Finland in 1980 and the Finnish Longitudinal Employer-Employee Data (FLEED) of Statistics Finland in 2001. The YFS began in 1980 when 4,320 participants from six age cohorts (aged 3, 6, 9, 12, 15 and 18) with the average age of 31.6 in 2001 and with the female share of 57.5. The participants were randomly chosen from five Finnish university regions based on the national population register [27]. In the baseline, a total of 3,596 persons participated in the YFS in 1980. Seven follow-up studies have been conducted since 1980, most recently in 2011/12.

Although men and younger participants have higher attrition rates in the Young Finns Study [27–28], analyses suggest this has no significant effect in earlier studies [27–28]. Data are collected using questionnaires, physical measurements and blood tests. All anthropometric measures of the YFS originate from professional health examinations conducted at local health centres.

The socioeconomic gradient in health is a potentially important confounder [26]. For this reason, we account for parental background. Therefore, we link the YFS to comprehensive register-based information from the LPC in 1980 using unique personal identifiers for parents and their children. To obtain additional controls for labor market success, the YFS data are linked to Statistic Finland’s Finnish Longitudinal Employer-Employee Data (FLEED). FLEED includes comprehensive information on individuals’ labour market status, and salaries and other income, taken directly from tax and other administrative registers that are collected and/or maintained by Statistics Finland.

### Ethical approval

We use previously collected data for which ethical approval was obtained. All YFS participants provided written informed consent, and the study was approved by local institutional review boards (ethics committees of the participating universities). Parents or guardians provided written informed consent on behalf of the under aged children enrolled in the study. The FLEED data have been constructed for research purposes by Statistics Finland, under the ethical guidelines of the institution which comply with the national standards. All data were anonymized and the study does not disclose information concerning individual persons. The use of linked YFS/FLEED data has been approved by Statistics Finland.

### Measures

We use the YFS to obtain information on happiness and biomarkers. Our dependent variable in the empirical specifications is a standard question on happiness in 2001. It is measured with a statement “In general, I feel happy” assessed on a 5-point scale (‘1’ = not agree; ‘5’ = agree).

Eight biomarkers used in the models as the variables of interest were obtained in 1980 when the participants were aged between 3 and 18 years old. There are four motivations for the inclusion of biomarkers that we examine in the paper. First, we use biomarkers that have been explored in an earlier contribution to the literature [29]. Second, biomarkers are related to cardiovascular diseases. The original aim of the YFS was to gather information that is relevant for cardiovascular risk. Third, we include only biomarkers that are available for the total population in the Young Finns Study. This is important in order to support statistical power. Fourth, in order to relate our work to earlier research using the YFS we use the same biomarkers as in earlier work [30].

Height (in millimeters) was measured by Seca anthropometer. We use information on triceps and subscapular skinfolds to calculate body fat based on the Slaughter equation [31]. Pulse was measured by listening to the heart beat directly and counting it for a minute. Blood pressure was measured from the brachial artery in sitting position after 5 minutes’ rest with a standard mercury sphygmomanometer. Serum total cholesterol and triglycerides were determined based on fasting venous blood samples after 12 hours of fasting. Serum samples were stored frozen at -20C for no more than 6 months. All lipid determinations were performed in duplicate in the same season (fall) and as simultaneously as possible and the averages of the two measurements were used in order to determine the level of serum lipids. Triglycerides were determined by using a fully enzymatic method (Boehringer Mannheim) [32]. Serum insulin was measured with a modification of the immunoassay method of Herbert et al. (1965) [33]. The amount of creatinine was measured using standard urine drug tests, based on the Jaffe method [34].

Our analyses start with the single measures from 1980 for four reasons. First, it makes sense to present evidence using single measures of biomarkers since, in most cases, that is all we have. Second, this is what would normally be presented in the literature. So showing the single measure estimates is useful when thinking about the rest of the literature. However, since we are in the fortunate position of having multiple measures for triglycerides (which were also collected in 1983, 1986 and 2001) we can establish the robustness of results to potential measurement error. Third, using the 1980 measures maximizes sample sizes for estimation. Fourth, the estimates using information on biomarkers in 1980 are less likely to suffer from residual confounding related to concurrent correlations.

An additional specification of the baseline model controls for educational attainment from FLEED in 2001. We use an indicator for those who have obtained tertiary education based on the highest obtained degree in 2001. We also control for register-based annual earnings from FLEED in 2001. We condition on family background using the family’s total annual income from LPC in 1980.

We also use relevant additional controls from YFS (1980). These covariates are not included in the baseline model because they are not available for the whole original sample that was gathered in 1980. Diet is a potentially important confounder in the relationship between biomarkers and SWB. For this reason, we control for fruit and vegetable consumption and the use of carbohydrates (starch, sucrose, lactose and other carbohydrates) in 1980 (measured as consumption per 1000 kcal). Since serum triglycerides are inversely associated with physical activity [35–36], and since we know that those who are more physically active are also happier [37], we control for physical exercise in childhood. Physical activity is measured with the question: “How often do you do sports (outside school hours)?” on a scale 1–7. Information on physical activity is not available for the two youngest cohorts in 1980.

Furthermore, individual differences in temperament and personality have been associated with happiness [38]. Thus, we used three temperament traits (negative emotionality, sociability and activity) as additional control variables. Details of their measurement have been reported elsewhere [39].

Table 1 provides descriptive statistics about the key variables. Raw correlations between the biomarkers are documented in Table 2. These indicate that pulse measured in 1980 was positively correlated with happiness in adulthood (2001), whereas triglycerides were negatively correlated.

**Table 1:** Descriptive statistics.

	Mean	SD	N
Happiness score on a scale 1–5 (2001)	4.219	0.858	1905
Height (1980)	142.045	25.746	1905
Body fat (1980)	17.394	7.466	1905
Pulse (1980)	80.926	14.693	1905
Systolic blood pressure (1980)	112.754	11.937	1905
Diastolic blood pressure (1980)	73.521	11.377	1905
Log of triglycerides (1980)	-0.322	0.323	1905
Insulin (1980)	9.635	5.844	1905
Log of creatinine (1980)	2.015	0.589	1905
High education (2001)	0.223	0.416	1905
Log of earnings (2001)	8.637	3.019	1905
Log of family income (1980)	9.326	0.643	1783
Fruit and vegetable consumption per 1000 kcal (1980)	162.567	110.678	945
Carbohydrates consumption per 1000 kcal (1980)	123.922	15.938	945
Physical activity on a scale 1–7 (1980)	5.487	1.486	1298

**Table 2:** Pairwise correlations between biomarkers and happiness.

	Height	Body fat	Pulse	Systolic	Diastolic	Log of triglyceride	Insulin	Log of creatinine	Happiness
Height	1								
Body fat	0.206***	1							
Pulse	-0.535***	0.010	1						
Systolic	0.521***	0.180***	-0.117***	1					
Diastolic	0.464***	0.115***	-0.268***	0.506***	1				
Log of triglyceride	0.183***	0.241***	-0.077***	0.141***	0.073***	1			
Insulin	0.581***	0.398***	-0.224***	0.370***	0.267***	0.329***	1		
Log of creatinine	0.574***	0.177***	-0.304***	0.282***	0.203***	0.091***	0.313***	1	
Happiness	-0.003	0.014	0.047**	0.005	0.009	-0.064***	-0.016	-0.012	1

Significant at ** 5% level.

Significant at *** 1% level.

### Statistical methods

The models are estimated using Ordinary Least Squares (OLS). All models include controls for sex, cohort and birth month. Triglyceride and creatinine were log-transformed because of skewed distributions. To assess the sensitivity of the baseline specification, we include additional controls for parental background and other relevant confounders. We also estimate separate models by gender and cohort. It is standard to use multiple regression analyses on sub-samples of the data when those sub-groups are not endogenously determined, as in the case of gender and cohort.

## Results

The baseline estimates (Column 1 of Table 3) show that only two out of eight biomarkers are statistically significant predictors of happiness. We find that pulse is positively related to happiness (2001). The quantitative magnitude of the effect of pulse on happiness implies that one standard deviation increase in pulse is associated with a 0.06 point increase in the happiness score on a scale of 1–5. The quantity of triglycerides measured in 1980 is also negatively related with happiness later in life, according to the baseline estimates. The relationship is statistically significant at the 1% level. The point estimate implies that one standard deviation increase in triglycerides is associated with a 0.05 point decrease in the happiness score. It is difficult to state whether the size of the triglyceride effect that we observe is small or not. The reason for this is that there are no earlier comparable estimates in the literature. Otherwise the baseline specification reveals that biomarkers measured in childhood are not significantly related to happiness in adulthood. It is interesting to note that there is no statistically significant relationship between height (1980) and happiness.

**Table 3:** The relationship between biomarkers (1980) and happiness (2001).

	(1)	(2)	(3)	(4)	(5)
Height	0.000	-0.001	0.001	-0.003	0.002
	(0.003)	(0.003)	(0.003)	(0.004)	(0.003)
Body fat	-0.005	-0.004	-0.003	-0.008	-0.003
	(0.004)	(0.004)	(0.004)	(0.006)	(0.005)
Pulse	0.004**	0.003**	0.004**	0.002	0.003
	(0.002)	(0.002)	(0.002)	(0.002)	(0.002)
Systolic blood pressure	0.001	0.001	0.001	0.001	0.002
	(0.002)	(0.002)	(0.002)	(0.003)	(0.003)
Diastolic blood pressure	-0.000	0.000	-0.000	-0.001	0.001
	(0.002)	(0.002)	(0.002)	(0.003)	(0.003)
Log of triglycerides	-0.169***	-0.162**	-0.138**	-0.236**	-0.150**
	(0.065)	(0.065)	(0.068)	(0.093)	(0.075)
Insulin	-0.003	-0.003	-0.005	0.006	-0.003
	(0.005)	(0.005)	(0.005)	(0.006)	(0.005)
Log of creatinine	-0.035	-0.038	-0.035	-0.005	-0.020
	(0.042)	(0.042)	(0.043)	(0.061)	(0.049)
Additional controls					
High education 2001		X			
Earnings 2001		X			
Family income 1980			X		
Fruit and vegetable consumption 1980				X	
Carbohydrates 1980				X	
Physical activity 1980					X
R^2^	0.0220	0.0348	0.0253	0.0311	0.0249
N	1905	1905	1783	945	1298

Biomarkers are measured in 1980. Dependent variable: subjective well-being measured with a statement “In general, I feel happy” assessed on a 5-point scale (‘1’ = not agree; ‘5’ = agree) in 2001. Heteroskedasticity-corrected robust standard errors are reported in parentheses. All models include controls for sex, cohort and birth month. Additional controls, as indicated. Significant at ** 5%

Significant at *** 1% level.

Columns 2–5 of Table 3 add extra controls to the baseline model. The negative relationship between triglycerides and happiness seems to be robust to the addition of various controls. The addition of earnings and educational attainment by adulthood in Column 2 tests for the possibility that biomarker effects on happiness may be mediated via their impact on educational and labour market behaviours. Although their inclusion increases the variance accounted for by the model the coefficients on the biomarker variables remain essentially unchanged.

Controlling for parental background in Column 3 using information from the LPC reduces the point estimate for triglycerides slightly (from -0.17 to -0.14), but the relationship remains intact and statistically significant at the 5% level. This result implies that the social environment of the child is not particularly important for the correlation between triglycerides and happiness.

Column 4 controls for fruit and vegetable intake and carbohydrate intake (starch, sucrose, and other carbohydrates) in childhood which might otherwise have been omitted variables linked to triglycerides which might have been driving their link to happiness. This proves not to be the case. Residual confounding due to diet is possible, because diet is notoriously difficult to measure accurately in survey data for two reasons. First, a large inventory would be needed to capture all possible aspects of food intake from an individual. Second, self-reported information on food intake suffers from recall bias and possibly from other forms of systematic measurement error that may bias the estimates.

Controlling for physical exercise in childhood has little impact on our results (Column 5). Thus, the negative correlation for triglycerides and SWB using the baseline measurement in 1980 is robust, based on the results that are reported in Table 3. However, we also observe that the finding for pulse is substantially more fragile and the result does not prevail after the addition of all potential controls to the baseline model (Columns 4–5).

We have also used three temperament traits (negative emotionality, sociability and activity) as additional control variables. Traits are measured in 1997. The result for triglycerides remains intact (not reported in Table 3). The coefficient for triglycerides in this model is -0.137. The standard error is larger (0.078), because the sample size is much smaller than in the baseline specification (n = 1210). Thus, the estimate was statistically significant only at the 10% level. The point estimate implies that one standard deviation increase in triglycerides is associated with a 0.01 point decrease in the happiness score.

There is very little evidence on the relationship between biomarkers and subjective well-being. For this reason, it makes sense to experiment with different specifications of the model. To assess the existence of a possible non-linear relationship between triglycerides and happiness, we added a quadratic term for triglycerides to the baseline specification of column 1 but this was not statistically significant. The non-significance of the quadratic triglycerides term implies that the parsimonious linear specification that is used in Table 3 is adequate to describe the relationship between triglycerides in childhood and adult happiness.

To establish how heterogeneous the relationships might be we ran sub-sample analyses but these reduce the sample size that is used in the estimates substantially. For this reason, we have to treat these results somewhat cautiously. We have run the estimates separately for men and women (not reported in Table 3). The relationship with triglycerides is statistically significant only for women. The coefficient for women is -0.222 with a robust standard error of 0.090. The estimate for men is -0.103 (0.096).

There is also a very notable difference in the relationship between age cohorts. The relationship between triglycerides and SWB prevails only for the three youngest age cohorts (n = 922). The coefficient for triglycerides for the three youngest age cohorts is -0.356 with a robust standard error of 0.101. The estimate for the oldest age cohorts is not statistically significant, not even marginally. The coefficient is -0.038 (0.085). The estimates for the specific subsamples using the baseline measurements suggest that the relationship between triglycerides and SWB is both gender- and age-specific.

Analyses involving biomarkers usually contain a single measurement on each biomarker. In Table 3 we use only the baseline measurements on biomarkers from 1980. But the YFS data contain measurements on triglycerides also in 1983, 1986 and 2001. This allows us to assess the robustness of the estimation results further and figure out whether the finding is spurious or not. The pairwise correlation coefficients within person between the baseline measurement of triglycerides in 1980 and the measurements in 1983, 1986 and 2001 are reported in Table 4. There are medium-size correlations between the measurements of triglycerides in 1980, 1983 and 1986 ranging from 0.391 to 0.512. All of the correlations are statistically significant at the 1% level. Table 4 also reports the correlations between triglyceride measures in the 1980s and in 2001. These correlations (between 0.294 and 0.385) show that there is medium-size persistence in the measurements of triglycerides from childhood to adulthood. Whilst significant throughout the size of the correlation coefficients depletes over time.

**Table 4:** Pairwise correlations for the triglyceride measures between 1980, 1983, 1986 and 2001.

	1980	1983	1986	2001
1980	1.000			
1983	0.508***	1.000		
1986	0.391***	0.512***	1.000	
2001	0.294***	0.350***	0.385***	1.000

Significant at *** 1% level.

We have estimated additional specifications using the measurements on biomarkers from 1983 and 1986. Information on creatinine was omitted from the analyses because it was available only in the 1980 sample. These results reveal that there is no statistically significant relationship between triglycerides and subsequent SWB in 2001 (Table 5). The baseline coefficient for triglycerides is -0.022 (with a robust standard error of 0.059) and 0.062 (0.070) using the measurements in 1983 and 1986, respectively. Compared to the estimates that are reported in Table 3, the conclusion about the non-existence of the relationship is not driven by the inflation of the standard errors. The point estimates also change substantially. To test further whether smaller sample size causes the statistical insignificance of the triglyceride estimates in the 1983 and 1986 samples we estimated the baseline model (Table 3, Column 1) using individuals who were both in the 1980 and 1983 (1986) samples. This reduces the sample size to 1556 (1334). Even with the smaller sample size the coefficients for triglycerides measured in 1980 remain significant (-0.148** for 1980/1983 sample and -0.182** for 1980/1986 sample) confirming that the insignificance of the later triglyceride measures is not driven by the sample size.

**Table 5:** The relationship between biomarkers (1983/1986) and happiness (2001).

	(1)	(2)	(3)	(4)
Height	-0.000	-0.002	0.000)	-0.000
	(0.003)	(0.003)	(0.000)	(0.000)
Body fat	-0.001	-0.001	-0.002	-0.000
	(0.004)	(0.004)	(0.004)	(0.004)
Pulse	0.002	0.002	-0.000	-0.000
	(0.002)	(0.002)	(0.002)	(0.002)
Systolic blood pressure	-0.003	-0.004	0.001	0.001
	(0.002)	(0.002)	(0.003)	(0.003)
Diastolic blood pressure	-0.000	-0.001	-0.006**	-0.006*
	(0.002)	(0.002)	(0.003)	(0.003)
Log of triglycerides	-0.022	-0.027	0.062	0.063
	(0.059)	(0.059)	(0.070)	(0.070)
Insulin	0.002	0.003	-0.002	-0.002
	(0.004)	(0.004)	(0.003)	(0.003)
Additional controls				
High education 2001		X		X
Earnings 2001		X		X
R^2^	0.0154	0.0265	0.0234	0.0354
N	1691	1691	1450	1450

In columns 1–2 and 3–4 the biomarkers were measured in 1983 and 1986, respectively. Dependent variable: subjective well-being measured with a statement “In general, I feel happy” assessed on a 5-point scale (‘1’ = not agree; ‘5’ = agree) in 2001. Heteroskedasticity-corrected robust standard errors are reported in parentheses. All models include controls for sex, cohort and birth month. Additional controls, as indicated.

Significant at *10% level.

Significant at ** 5% level.

Because the relationship between triglycerides and SWB prevails only for the three youngest age cohorts using the baseline measurement in 1980, we have estimated the models also separately for the youngest three age cohorts in 1983 and 1986. The coefficient for triglycerides is -0.071 (with a robust standard error of 0.087) and 0.055 (0.101) using the measurements in 1983 and 1986, respectively. Based on these results using the later measurements on triglycerides, we conclude that the relationship between triglycerides and subsequent SWB is not robust in the sense that the negative correlation is only apparent using the baseline measurement in 1980. Using the other two early triglyceride measures (1983/1986) we find no link to adult well-being (Table 5).

Finally, we cannot estimate exactly the same models as those in Table 3 using the full set of biomarkers in adulthood. However, after adjusting for gender, cohort and birth month the measure of triglycerides in 2001 is negatively correlated with SWB in 2001 but the correlation is not statistically significant (coefficient -0.046, robust standard error 0.044). Concurrent correlations are plagued by confounders. For this reason, we use the longitudinal aspect of the data and measure biomarkers in 1980 (and 1983/1986) and SWB in 2001.

## Discussion

Using longitudinal research design and nationally representative data for a cohort of young Finns we find that biomarkers in childhood are not robustly associated with adult happiness while controlling for the key non-biological markers. We do find that there is a negative relationship between triglycerides and subjective well-being but it is both gender- and age-specific and the relationship does not prevail using the later measurements (1983/1986) on triglycerides. The standard approach in the literature is to use single measurements on biomarkers. Given the potential for biomarkers to be measured with error and/or for biomarkers to vary (even within the day), the correlations with other factors can change over time.

Using linked data we account for a very rich set of confounders including family and socio-economic background factors. Biomarkers and happiness could be co-determined by a third factor, such as genetic predispositions, which are unaccounted in most data sets that are available. Unobserved traits related to genetic endowment are potentially very important. De Neve et al. [40] show that around one-third of variation in life satisfaction is explained by genetic variation.

Our results show that it is important to use multiple measurements on biomarkers. We found a robust relationship using a single measurement of triglyceride in the blood recorded in childhood. However, triglyceride levels can be determined at any point in time by proximate factors, such as food intake, which might temporarily boost or reduce triglyceride levels, perhaps giving an unreliable reading of underlying triglyceride rates. This measurement problem should reduce the likelihood of finding any statistically significant association between biomarkers in childhood and happiness in adulthood. On the other hand, relying on a single measurement point may easily lead to spurious conclusions that do not extend to multiple measurements on biomarkers.

It is also possible that happier people function or behave in such a way as to induce changes in their biomarker readings in a way that we cannot observe. We know, for example, that happier people behave in healthier ways, for instance with respect to food intake [41]. Whether this explanation is relevant for young people remains an open question.

There seems to be little association between other childhood biomarkers and happiness in adulthood. It might not have been surprising to find an association between height and happiness since height is associated with higher earnings [42] and others’ perceptions of one’s attractiveness, both of which one might expect to be linked to happiness. Body fat can affect happiness through one’s self-perception, while blood pressure is a potential marker for poor health. But none of these factors were predictive of adult happiness. Pulse measured in childhood was positively associated with adult happiness in three of our five equations but the correlation became statistically non-significant with the introduction of childhood fruit and vegetable consumption, confirming what others have found about the importance of nutrition for psychological well-being [43]. Future research is warranted to see if the poor prediction power of biomarkers regarding happiness can be replicated for other populations. Also, it is possible that there are some other biomarkers that are significantly related to happiness later in life that were not included in our study.

To summarize, our analysis examining long-term predictors of happiness over a twenty-year period shows that none of the eight biomarkers measured in childhood predict happiness robustly in adulthood. In this sense, happiness is not determined by biology.
